# The Outcome of Autologous Platelet-Rich Fibrin Membrane in Failed Distal Hypospadias Repair

**DOI:** 10.5152/tud.2025.25049

**Published:** 2025-12-05

**Authors:** Khaled M. Abdelhalim, Esam Abdelgawad, Ahmed M. Kadry, Mohamed Bakr, Hassan Abdelwahab

**Affiliations:** Department of Urology, Suez Canal University Faculty of Medicine, Ismailia, Egypt

**Keywords:** Autologous platelet-rich fibrin, failed distal hypospadias repair, outcome

## Abstract

**Objective:**

Failed distal hypospadias repair is considered a challenge due to the deficient scarred local penile tissue for tubularization and neourethral coverage in tubularized incised plate (TIP) repair and the high complications rate. To assess the additive effect of autologous platelet-rich fibrin (PRF) membrane neourethral coverage layer on failed distal hypospadias TIP repair outcomes.

**Methods:**

**:** A total of 66 recurrent distal hypospadias patients who were suitable for TIP repair were divided into group A: 32 patients in whom a local penile dartos flap layer was used and group B: 34 patients in whom an autologous PRF membrane was used. All patients were followed up for 6-12 months postoperatively for the presence of any complications.

**Results:**

The reoperation rate in the Dartos group was more than twice that observed in the PRF group (28.1% vs. 11.8%; *P* = .11). Similarly, the rate of meatal stenosis (12.5% vs. 2.9%; *P* = .18), infection (15.6% vs. 8.8%; *P* = .46), and dehiscence (3.1% vs. 2.9%; *P* = 1.00) were all higher in the Dartos group. Urethrocutaneous fistula was reported in 7 patients, with 6 (18.75%) in group A and 1 (2.9%) in group B, showing a statistically significant difference (*P* < .05).

**Conclusion:**

The autologous PRF membrane could be an effective neourethral coverage layer in comparison to local dartos flap in decreasing complications rate post failed distal hypospadias TIP repair.

## Introduction

Different techniques for failed distal hypospadias have been utilized, with ongoing advancements and modifications emerging continuously. Tubularized incised plate (TIP) repair has achieved broad approval for the correction of failed distal hypospadias.^1^ The urethrocutaneous fistula (UCF) incidence ranges from 3% to 10% following primary hypospadias surgery.^2^

Risk of complications is twice as likely with a single failed hypospadias urethroplasty as it is with the first repair; the risk rises to 40% with 3 or more reoperations. This finding lends credence to the idea that penile tissue vascularity declines with each surgery and points to the necessity of therapies aimed at restoring vascularity.^3^

Fistulas and other complications are significantly reduced when a protective barrier is placed between the neourethra and the skin. In order to avoid UCF, many second-layer covering layers have been detailed, which include either local penile subcutaneous tissue, tunica vaginalis, dartos, or extragenital tissues such as a free skin graft.^4^

The use of local flaps is limited in recurring instances due to excessive scar formation and reduced blood supply to neighboring tissues. As a result, additional tissues with greater wound-healing properties must be used.^2^

The platelet-rich fibrin (PRF) offers numerous benefits in comparison to other fibrin sealants. It is easy to produce and is entirely derived from the patient’s own serum. Consequently, the risk of infection, allergic reactions, and the negative effects of a high fibrinogen concentration are all eliminated.^5^^,^^6^

The growth factors that PRF mediates, such as transforming growth factor-β, platelet-derived growth factor, insulin-like growth factor-1, epidermal growth factor, and vascular endothelial growth factor, are abundant in PRF. Therefore, PRF speeds up the healing process. In addition, PRF has a high concentration of leukocytes and other host immune cells that aid in wound healing and infection resistance.^7^

This study was designed to assess the outcome of autologous PRF membrane neourethral coverage in failed distal hypospadias TIP repair.

## Material and Methods

This was a prospective randomized study conducted on 66 patients with failed distal hypospadias at a single center. Approval was obtained from the Ethics Committee under number 5069/2022. The parents provided informed consent.

### Patients’ Population

Inclusion criteria included failed circumcised distal penile hypospadias repair patients (1 primary urethroplasty trial) who were candidates for TIP repair. A narrow secondary urethral plate of less than 8 mm, disfigured glans, and deficient scarred local penile skin were excluded from the study.

The sample size was calculated utilizing the equation of the variance between 2 means (mean and standard deviation):^8^ n = 2 {(Zα + Zβ) σ / µ1 − µ2}^2^

Where: n: sample size per group, Zα: The value of standard normal distribution for type І error probability for a 2-sided test (0.05/2) = 1.96, Zβ: The value of standard normal distribution for the desired statistical power (95%) = 1.645, µ1 − µ2: The study complications difference between the first and second groups = 26 − 0 = 26.^9^ σ: The within-group standard deviation = 25.69. The final sample size was 20 cases per group with an additional 20% dropout in each group. Sixty-six patients were randomized into 2 groups using randomly generated numbers by the random allocation software (Sealed Envelope Allocation Software) (version 2.3).^10^

Personal history, present history, family history, past operative history, and genital examination, including external urethral meatus site and diameter, glanular shape, residual penile curvature, penile torsion, secondary urethral plate width (at glans point), and availability and condition of local penile skin, were recorded in all patients.

The primary objective was to assess the outcome of autologous PRF membrane neourethral coverage layer in reoperative distal hypospadias TIP repair.

### Surgical Technique

**Figure 1.**
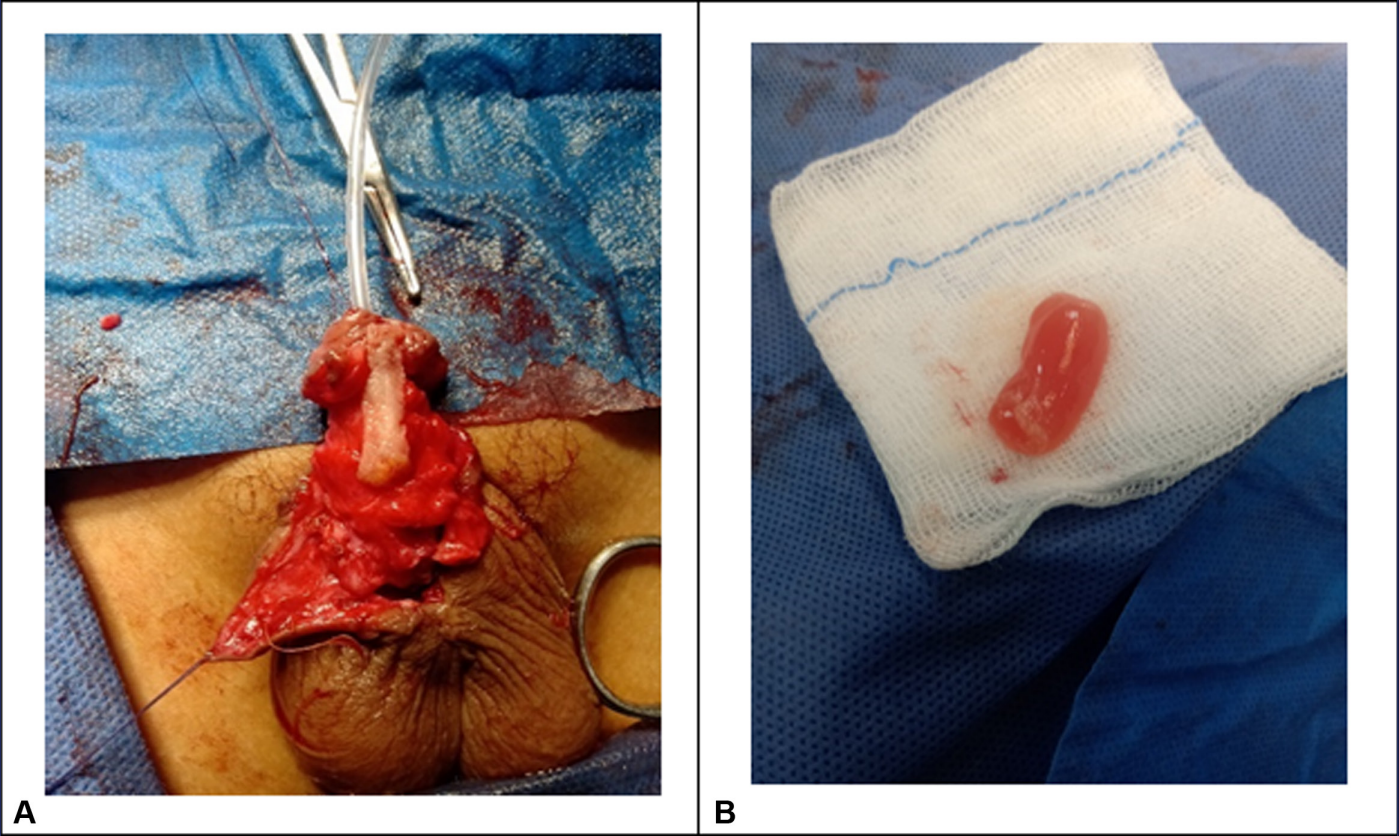
(A) PRF membrane neourethral coverage during TIP repair. (B) PRF membrane 10-15 mm in length and 1 mm in thickness.

Surgeries were performed by a single surgeon using the same TIP repair and the same suture materials. A U-shaped incision was made outside the apparent edges of the secondary urethral plate, 2 millimeters proximal to the original meatus. A deep, relaxing midline incision was performed to broaden the urethral plate, then tubularized over a silicone catheter 6-10 Fr, starting proximally and moving distally. The first layer closure was done with interrupted 6/0 polyglactin subcuticular sutures, terminating before the glans tip, rendering an oval neomeatus with an appropriate diameter. After that, the target population was divided into 2 groups according to the neourethral coverage layer: group A: 32 patients in whom a local penile dartos flap layer was used and group B: 34 patients in whom an autologous PRF membrane was used and secured by polygalactin 6/0 (Figure 1A). After that, closure of the glanular wings and penile shaft skin was done with 4/0 polyglactin sutures.

### Intraoperative Preparation of the Platelet-Rich Fibrin Membrane

About 10 mL of blood must be collected from the patient without anticoagulant in a glass or glass-coated plastic tube as part of the PRF production protocol. The blood was promptly centrifuged at 2700 rpm for 12 minutes using a table centrifuge (Thermo Scientific Medifuge™ 2017, USA) as soon as it was collected.^11^ After the cycle is finished, the blood in the tube is separated into 3 distinct layers: platelet-poor plasma at the top, PRF in the middle, and a red blood corpuscular foundation at the bottom. Forceps were employed to meticulously extract the PRF clot from the test tube, and the RBC base was meticulously removed to preserve a small portion of it within the clot. The clot that is subsequently obtained can be gently compressed between 2 surgical probes to produce a flexible and resistant membrane that is approximately 10-15 mm in length and 1 mm in thickness. The process was carried out under strict aseptic conditions without any additional cost except of price of the sterile collecting tube and centrifuge device.^12^ (Figure 1B).

### Postoperative Evaluation

A silicone urethral stent was kept for 10 days and removed in the outpatient clinic. Penile dressing was removed on the fifth day postoperatively, and the wound was left exposed for daily saline 0.9% and Betadine dressing only until catheter removal. All patients received parenteral ampicillin/sulbactam, starting intraoperatively and postoperatively for 2 days, followed by oral cefixime until urethral catheter removal.

The parents or patients were asked about the urine stream’s quality, caliber, and whether there was any voiding difficulty or leakage during the follow-up examination. All patients were assessed at 1 week, 1 month, and 6 and 12 months postoperatively for follow-up of the presence of any of the following complications: presence of hematoma, presence of wound infection, wound dehiscence by inspection, presence of UCF, meatal stenosis, and urethral stricture by inspection, Nelaton catheter calibration, or uroflowmetry in older toilet-trained boys.

Successful outcome was defined as no reported postoperative complications or only minor complications that were treated conservatively. Failed outcome was defined as the occurrence of postoperative complications that needed immediate intervention or later secondary auxiliary intervention after 6 months.

### Data Analysis

The data entry will be via SPSS™. Mainly, the comparison of the study variables will be via detecting the significance of differences between mean values of each parameter using chi-square or student’s *t*-test, with significance set at a *P*-value of <.05. Odds ratio and relative risk were calculated for surgical outcomes between the studied group variables. Finally, the data will be arranged into tables to conclude the findings of the study. No multivariate analysis was used in this study.

## Results

**Figure 2.**
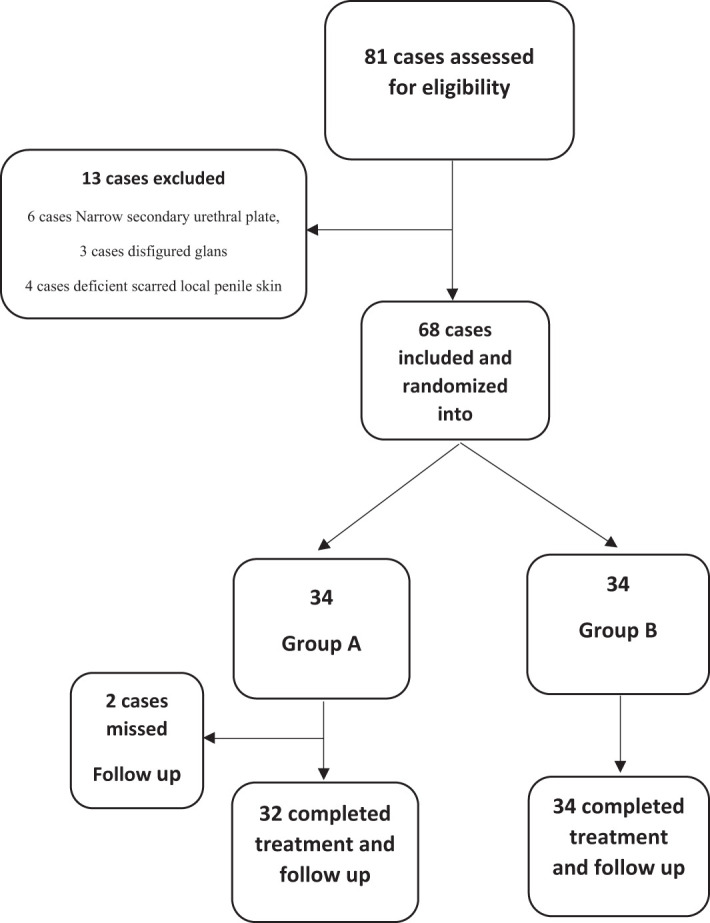
Flowchart of study.

The study assessed 81 patients with hypospadias, with only 68 patients meeting inclusion criteria and undergoing surgery. Two patients from group A were lost to follow-up, resulting in 32 patients, with 34 patients in group B (Figure 2).

There were 66 patients in the study; 28 (42.4%) were coronal, 17 (25.8%) were subcoronal, and 21 (31.8%) were distal penile. The 2 groups were identical and comparable in terms of demographic data and preoperative clinical presentation. They were randomly divided into 2 groups (Table 1). Their average age at surgery was 45.7 months, and it varied from 20 to 89 months. All patients had no additional genitourinary abnormalities (Table 1).

Group A had a significantly shorter mean operative time (102.8 minutes) compared to group B (107.9 minutes) (*P* < .05). The UCF was reported in 7 patients, with 6 (18.75%) in group A and 1 (2.9%) in group B, showing a statistically significant difference (*P* < .05) (Table 2).

In Table 3, Dartos flap group showed higher rates of complications compared to PRF across all surgical outcomes; however, none of the observed differences was statistically significant. The reoperation rate in the Dartos group was more than twice that observed in the PRF group (28.1% vs. 11.8%; *P* = .11). Similarly, the rate of meatal stenosis (12.5% vs. 2.9%; *P* = .18), infection (15.6% vs. 8.8%; *P* = .46), and dehiscence (3.1% vs. 2.9%; *P* = 1.00) were all higher in the Dartos flap group.

## Discussion

The fundamental principles of any surgery remain unchanged, despite the advancements in surgical procedures for the correction of hypospadias: deliver a patient with a terminal or near-terminal meatus and a straight penis that is visually and functionally acceptable with minimal morbidity.^13^

The type of hypospadias, suturing technique, and post-operative care have all been cited as variables that may influence the outcome of TIP hypospadias repair.^14^ To decrease the risk of fistulas in TIP, the most important step is to avoid crossing suture lines thus placing interposition tissue over the neourethra is a must. After TIP repair, fistulas were found in up to 30% of patients lacking a second layer.^15^ Several studies have studied and proven the value of multi-layer neo-urethral covering. Some suggested a second layer of tunica vaginalis flap.^2^^,^^16^^,^^17^

The PRF, a second generation of platelet concentrates, is gaining popularity as a biologic adjunct to surgical repairs. The most extensively used PRF is not only significantly more physiologically effective than its predecessors, but it is also much simpler and less complex.^1^

In this study, in terms of operative time, hospital stays, and complications, group A had a considerably shorter mean operative time (102.8 minutes) than group B (107.9 minutes) (*P* < .05). The UCF was reported in 7 patients, with 6 (18.75%) in group A and 1 (2.9%) in group B, indicating a significant distinction. The reoperation rate in the Dartos group was more than twice that observed in the PRF group (28.1% vs. 11.8%; *P* = .11). Similarly, the rate of meatal stenosis (12.5% vs. 2.9%; *P* = .18), infection (15.6% vs. 8.8%; *P* = .46), and dehiscence (3.1% vs. 2.9%; *P* = 1.00) were all higher in the Dartos flap group.

The effectiveness of an autologous PRF membrane in improving healing and reducing fistula development in patients with primary distal hypospadias was assessed by Abdelazim et al.^18^ Forty patients suffering from primary distal hypospadias were included in the research. Using a computer-based randomization system, eligible patients were divided into 2 groups: one that received a dartos flap as a first covering layer over the repair (group A) and another that received PRF (group B). The average age at surgery for group A was 27.1 ± 17.7 months (which varied from 6 to 61), whereas for group B it was 24.8 ± 19.2 months (which varied from 6 to 67). The same surgeon performed all of the TIP urethroplasty procedures.

In accordance with Abdelazim et al^18^, 7 cases of fistula in group A needed surgical correction after 6 months of follow-up; however, no recurrence of fistula was seen in any of the cases at the end of the follow-up period. Although 1 patient in group B (5% of the total) developed meatal stenosis, 2 individuals in group A (10%) did so. One patient (5% of the total) in group B experienced glandular dehiscence.

The additive effect of PRF usage as neourethral coverage in primary distal hypospadias TIP repair was assessed by Mansour et al^19^ who compared the types of coverage layer in TIP repair in group A of a single dartos flap only with group B of PRF plus dartos flap.

In agreement with Mansour et al^19^ who found that group B had the lowest rate of complicated cases (9.1% compared to 31% for group A) without statistical significance, the implementation of PRF in group B scaled down the rate of UCF formation to 4.5% compared to 18.2% in group A. Furthermore, group B had no incidence of wound infection compared to 22.7% in group A.

Platelet-rich plasma (PRP) has been studied for its efficacy in hypospadias correction. Eryilmaz et al^20^ used PRP gel to increase coverage in mid-penile hypospadias repair, resulting in a 10% UCF, 5% urethral stenosis, and 5% wound infection rate in the PRP group. In comparison, the group without PRP had 25% UCF, 25% urethral stenosis, and 35% wound infection rates.

Also, concurred with Wishahy et al^21^, who did research on 37 patients to examine the efficacy of PRF in cases with UCF; 20 patients received local dartos covering and 17 received PRF. They reported that there was no significant improvement in the results after applying the PRF membrane; however, there was a decrease in the incidence of recurrent fistulas after applying PRF, which may necessitate more cases to detect a significant variation among the 2 techniques. No control group was included in their study.

In single-arm research, Al-Awadi et al^22^ examined only recurring or circumcised instances, as a healthy preputial dartos is not present. Although recurrent cases are expected to have a higher risk of complications (up to 30%), their UCF rate of 6.7% is comparable to ours and 10% with Elsayed et al. Additionally, Elsayed et al. and Al-Awadi et al^22^^,^^23^ found just 1 case of wound infection in their research.

The current study found that the reoperation rate in the Dartos group was more than twice that observed in the PRF group (28.1% vs. 11.8%). Group A had 9 out of 32, while group B had 4 out of 34, with a statistically insignificant difference (*P* = .11).

In group A, there were 6 cases of UCF (associated with meatal stenosis in 2 cases and wound infection in 4 cases), 1 case of glans dehiscence, and 2 cases of meatal stenosis which were treated by reoperation after 6 months without reported other complications. In addition, there was 1 case of mild wound infection and another case of mild hematoma which were managed conservatively without further complications. In group B, there was 1 case of UCF, 1 case of meatal stenosis, and 1 case of glans dehiscence which were managed by reoperation after 6 months without reported complications, as well as 1 case of wound infection which was managed by immediate drainage without reporting other further complications. Additionally, there were 2 cases of mild wound infection which were managed conservatively without complications.

Despite the study’s strength being prospective and randomized, it contained only failed circumcised distal hypospadias repairs and was performed using the same procedure and surgeon to avoid result bias. There were several limitations, such as a short follow-up time, as well as the need for a multicenter study design to assess the efficacy of PRF membrane in enhancing wound healing. Additionally, no histological comparison was performed between groups with and without PRF to evaluate in vivo postoperative tissue changes after PRF application.

The autologous PRF membrane could be an effective neourethral coverage layer in comparison to local dartos flap in decreasing complications rate post failed distal hypospadias TIP repair.

## Figures and Tables

**Table 1.** Demographic Data of Studied Groups

			Dartos Flap	PRF	Total	*P*
Meatus location	Coronal		15	13	28	.752^C^
	Subcoronal		8	9	17	
	Distal penile		9	12	21	
Secondary urethral plate shape	Shallow		19	16	35	.316^C^
	Deep grooved		13	18	31	
Secondary urethral plate width	8 mm		18	16	34	.455^C^
	>8 mm-10 mm		14	18	32	
Glans circumference	15 mm		17	18	35	.988^C^
	>15 mm		15	16	31	
Chordee	No chordee		14	14	28	.833^C^
	<30 degrees		18	20	38	
Penile torsion	No torsion		24	21	45	.249^C^
	<30 degrees		8	13	21	
Glans shape	Groove		15	11	26	.228^C^
	Flat		17	23	40	
Sufficient local penile skin condition	Pliable		24	23	47	.51^C^
	Scared		8	11	19	
	**n**		**Mean**	**Standard Deviation**	**Standard Error Mean**	***P***
Age (months)	Dartos flap	32	47.78	24.548	4.340	.458^T^
	PRF	34	43.76	18.961	3.252	
Timing from previous operation (months)	Dartos flap	32	12.13	5.308	0.938	.068^T^
	PRF	34	15.06	7.315	1.255	
Meatus size (Fr)	Dartos flap	32	7.50	0.880	0.156	.517^T^
	PRF	34	7.35	0.950	0.163	

C, Chi test; T, *t*-test.

**Table 2.** Operative Time, Hospital Stay, and Complications

		n	Mean	Standard Deviation	Standard Error Mean	*P*
Operative time (minutes)	Dartos flap	32	102.81	9.972	1.763	.039^T*^
	PRF	34	107.91	9.721	1.667	
Hospital stay (days)	Dartos flap	32	4.81	1.575	.278	.0978^T^
	PRF	34	4.82	1.678	.288	
Complications			**Dartos flap**	**PRF**	**Total**	***P***
Urethrocutanous fistula	Yes		6	1	7	.037^C^
	No		26	33	59	
Meatal stenosis	Yes		4	1	5	.142^C^
	No		28	33	61	
Infection	Yes		5	3	8	.397^C^
	No		27	31	58	
Glans dehiscence	Yes		1	1	2	.965^C^
	No		31	33	64	
Hematoma	Yes		1	0	1	.299^C^
	No		31	34	63	
Postoperative chordee	Yes		0	0	0	N/A
	No		32	34	66	
Postoperative penile torsion	Yes		0	0	0	N/A
	No		32	34	66	

C, Chi test; T, *t*-test.

* Significant.

**Table 3.** Surgical Outcome of 2 Studied Groups

	Dartos Flap (n = 32) N (%)	PRF (n = 34) N (%)	Relative Risk (95% CI)	*P* ^1^
Reoperation rate	9 (28.1)	4 (11.8)	0.418 (0.142-1.22)	.11
Meatal stenosis	4 (12.5)	1 (2.9)	0.235 (0.027-1.99)	.18
Infection	5 (15.6)	3 (8.8)	0.564 (0.146-2.17)	.46
Hematoma	1 (3.1)	0 (0)	0.314 (0.013-7.44)	.48
Dehiscence	1 (3.1)	1 (2.9)	0.94 (0.061-14.4)	1.00

^1^Fisher’s exact test.

## Data Availability

The data that supports the findings of this study is available on request from the corresponding author.

## References

[1] Snodgrass WT Bush N Cost N. Tubularized incised plate hypospadias repair for distal hypospadias. J Pediatr Urol. 2010;6(4):408 413. (doi: 10.1016/j.jpurol.2009.09.010) 19837000

[2] Fahmy O Khairul-Asri MG Schwentner C Algorithm for optimal urethral coverage in hypospadias and fistula repair: a systematic review. Eur Urol. 2016;70(2):293 298. (doi: 10.1016/j.eururo.2015.12.047) 26776935

[3] Snodgrass W Bush N. Staged tubularized autograft repair for primary proximal hypospadias with 30-degree or greater ventral curvature. J Urol. 2017;198(3):680 686. (doi: 10.1016/j.juro.2017.04.019) 28400187

[4] Staninec M Darling CL Goodis HE Pulpal effects of enamel ablation with a microsecond pulsed λ= 9.3-µm CO2 laser. Lasers in surgery and medicine. Lasers Surg Med. 2009;41(4):256 263. (doi: 10.1002/lsm.20748) 19347946 PMC3188421

[5] Huber SC Cunha JL Montalvo SAL In vitro study of the role of thrombin in platelet rich plasma (PRP) preparation: utility for gel formation and impact of growth factors release. J Stem Cells Regen Med. 2016;12:2 9. 27397996 10.46582/jsrm.1201002 PMC4929890

[6] Reis CHB Buchaim DV Ortiz AC Application of fibrin associated with photobiomodulation as a promising strategy to improve regeneration in tissue engineering: a systematic review. Polymers. 2022;14(15):3150. (doi: 10.3390/polym14153150) PMC9370794 35956667

[7] Miron RJ Zhang Y. Autologous liquid platelet rich fibrin: a novel drug delivery system. Acta Biomater. 2018;75:35 51. (doi: 10.1016/j.actbio.2018.05.021) 29772345

[8] Dawson B Trapp RG. Basic and Clinical Biostatistics. 4th ed. USA: McGraw-Hill’s access medicine; 2004:13.

[9] Soyer T Ayva ES Atasoy P Comparison of growth factor levels in patients with normal and hypospadias prepuce. Turk J Med Sci. 2011;41:81 85.

[10] Aziz MJ Gayme DF Johnson K A co-design framework for wind energy integrated with storage. Joule. 2022;6(9):1995 2015. (doi: 10.1016/j.joule.2022.08.014)

[11] Dohan DM Choukroun J Diss A Platelet-rich fibrin (PRF): a second-generation platelet concentrate. Part I: Technological concepts and evolution. Oral Surg Oral Med Oral Pathol Oral Radiol Endod. 2006;101(3):e37 e44. (doi: 10.1016/j.tripleo.2005.07.008) 16504849

[12] Guinot A Arnaud A Azzis O Habonimana E Jasienski S Frémond B. Preliminary experience with the use of an autologous platelet-rich fibrin membrane for urethroplasty coverage in distal hypospadias surgery. J Pediatr Urol. 2014;10(2):300 305. (doi: 10.1016/j.jpurol.2013.09.026) 24325905

[13] Castagnetti M El-Ghoneimi A. Surgical management of primary severe hypospadias in children: an update focusing on penile curvature. Nat Rev Urol. 2022;19(3):147 160. (doi: 10.1038/s41585-021-00555-0) 35039660

[14] Aziz MS. Hypospadias repair: surgical management and post operative assessment. Tobacco Regulatory Science (TRS). 2023:6119 6140.

[15] Mbouche LO Mbassi AA Mekeme JBM Characteristics and management of post-circumcision urethrocutaneous Fistula: a retrospective study in surgical units in Cameroon. BJUI Compass. 2024;5(7):681 690. (doi: 10.1002/bco2.391) 39022657 PMC11250727

[16] AbouZeid AA Habak RA Hamad MM Shahin AM. De-epithelialized overlap flap to secure urethroplasty in second stage hypospadias repair: revisiting the Smith technique. BMC Urol. 2023;23(1):143. (doi: 10.1186/s12894-023-01312-8) PMC10469420 37648994

[17] Safwat A Al-Adl AM El-Karamany T. Vascularized dartos flap in conjunction with tubularized incised plate urethroplasty: single versus double flaps for management of distal hypospadias. Curr Urol. 2012;6(2):67 70. (doi: 10.1159/000343511) 24917716 PMC3783336

[18] Abdelazim O Abdullateef KS Khedr E Tarek M. The use of an autologous platelet-rich fibrin membrane in urethroplasty for cases of distal hypospadias. Egypt Pediatric Association Gaz. 2024;72(1):58. (doi: 10.1186/s43054-024-00304-z)

[19] Mansour AM Ismail EA Abdalla MO El Nashar AM Ismail IY Abdelhalim KM. Additive outcome of platelet rich fibrin neourethral coverage of tubularized incised plate in primary distal hypospadias repair. BMC Urol. 2024;24(1):265. (doi: 10.1186/s12894-024-01591-9) PMC11629491 39658803

[20] Eryilmaz R Şimşek M Aslan R Beger B Ertaş K Taken K. The effect of plasma rich platelet graft on post-operative complications in mid-penile hypospadias. Andrologia. 2020;52(7):e13652. (doi: 10.1111/and.13652) 32436309

[21] Wishahy AM Abdullateef KS Kaddah SN Mohamed AA Mohamed MT. Surgical evaluation of autologous platelet-rich fibrin membrane as a coverage layer in repair of urethrocutaneous fistula after hypospadias surgeries: a randomized controlled trial. J Indian Assoc Pediatr Surg. 2024;29(5):505 510. (doi: 10.4103/jiaps.jiaps_149_22) 39479420 PMC11521221

[22] Fouad Al-Awadi AS Megahed HA Ahmed Shahin MM Abdul Aziz FA. The use of autologous platelet rich fibrin membrane as a second layer in Snodgrass repair of distal hypospadias. Int J Med Arts. 2021;3(2):1377 1383. (doi: 10.21608/ijma.2021.44160.1181)

[23] El-Sayed IM Moustafa WA El-Zamarany EA Sadaka MS. The use of autologous platelet-rich fibrin membrane in hypospadias surgery: a preliminary study. Tanta Med J. 2017;45(4):161 165. (doi: 10.4103/tmj.tmj_48_17)

